# Bidirectional shifts in *Pm20d1* expression impact thermogenesis and metabolism

**DOI:** 10.1186/s10020-025-01345-9

**Published:** 2025-08-28

**Authors:** Marcela R. Simoes, Ana L. Gallo-Ferraz, Bruna Bombassaro, Fernando Valdivieso-Rivera, Guilherme A. S. Nogueira, Milena Monfort-Pires, Marcos Vinicius da Cruz, Ariane M. Zanesco, Nayra Fernanda-Oliveira, Leonardo Reis Silveira, Roger F. Castilho, Carlos H. Sponton, Licio A. Velloso

**Affiliations:** 1 Laboratory of Cell Signaling - Obesity and Comorbidities Research Center, University of Campinas (UNICAMP), Campinas, SP 13083-864 Brazil; 2 Department of Physiology, Ribeirão Preto Medical School, University of São Paulo, Ribeirão Preto, SP Brazil; 3 Turku PET Centre, University of Turku, Turku, 20520 Finland; 4 Department of Structural and Functional Biology, Institute of Biology (IB), University of Campinas (UNICAMP), Campinas, 13083-862 SP Brazil; 5 Department of Pathology, School of Medical Sciences, University of Campinas (UNICAMP), Campinas, 13083-888 SP Brazil; 6 National Institute of Science and Technology on Neuroimmunomodulation, Campinas, 13083-864 SP Brazil

**Keywords:** Fatty acid, Lipid, Mitochondria, Thermogenesis, Obesity

## Abstract

**Background:**

Peptidase M20 domain containing 1 (PM20D1) is a secreted N-fatty acyl amino synthase and hydrolase that controls tissue and blood levels of N-fatty acyl amino acids. In brown adipocytes, N-fatty acyl amino acids bind to mitochondria and act as uncouplers of mitochondria, independent of UCP1. Interventions aimed at increasing or inhibiting PM20D1 expression considerably impact energy balance and metabolism; however, little is known about naturally occurring variants of the *PM20D1/Pm20d1* gene and their impact on phenotype.

**Methods:**

In vivo, gene expression of *Pm20d1* in BALB/c, C57BL/6, and *Ucp1* KO in brown adipose tissue and other metabolic tissues was measured. In vitro, transcriptional activity of *Pm20d1* and brown adipocytes’ oxygen consumption in primary culture were assessed. Human PM20D1 circulating levels were quantified. In silico analysis of the *Pm20d1* gene sequencing and human polymorphisms associated with *PM20D1* was performed.

**Results:**

Here, we identified a gain-of-function variant in the *Pm20d1* promoter region present in BALB/c mice and absent in C57BL/6 mice. The presence of this variant is accompanied by increased expression of *Pm20d1* in brown and white adipose tissues, muscle, liver, and hypothalamus; moreover, it leads to increased cold tolerance and UCP1-independent brown adipose tissue mitochondrial respiration. Inhibition of *Pm20d1* in brown adipose tissue results in defective cold tolerance in BALB/c, whereas the brown adipose tissue overexpression of *Pm20d1* results in increased cold tolerance in C57BL/6 mice. In humans, variants of the *PM20D1* gene are associated with changes in body mass index, whereas at least one variant in the promoter region is associated with increased body mass index and metabolic syndrome.

**Conclusion:**

Thus, PM20D1 plays a bidirectional role in regulating thermogenesis and body mass, and, at least in part, variants in the promoter region can partially explain the differences in PM20D1 expression and its impact on the metabolic phenotype.

**Supplementary Information:**

The online version contains supplementary material available at 10.1186/s10020-025-01345-9.

## Introduction

The identification of active brown adipose tissue (BAT) in adult humans (Cypess et al. (Cypess et al. 2009) (Virtanen et al.(Virtanen et al. 2009) has stimulated the search for interventions that could promote thermogenesis as an approach to increase energy expenditure and the uptake and oxidation of glucose and fatty acids (Carpentier et al.(Carpentier et al. 2023). The canonical mechanism of BAT thermogenesis depends on the activity of uncoupling protein 1 (UCP1), which utilizes the electrochemical gradient across the mitochondrial inner membrane to dissipate energy as heat instead of producing ATP (Fedorenko et al.(Fedorenko et al. 2012) (Nicholls(Nicholls 2021) (Cannon and Nedergaard(Cannon and Nedergaard 2004). However, as*Ucp1* knockout (KO) mice retain some capacity of activating uncoupled mitochondrial respiration and thermogenesis (Ikeda and Yamada (Ikeda and Yamada 2020), it has been proposed that mechanisms other than UCP1 could exist. In fact, research carried out over the last 10 years has led to the identification of at least three distinct UCP1-independent mechanisms: (i) a futile creatine-driven substrate cycle that consumes ATP via creatine kinase activity (Kazak et al.(Kazak et al. 2015); (ii) sarcoplasmic/endoplasmic-reticulum calcium cycling (Ikeda et al.(Ikeda et al. 2017); (iii) and, the activity of peptidase M20 domain containing 1 (PM20D1) (Long et al.(Long et al. 2016).

Whereas great advances have been obtained in the understanding of the UCP1-independent mechanisms driven by creatine kinase and sarcoplasmic/endoplasmic-reticulum calcium cycling (Kazak and Cohen (Kazak and Cohen 2020) (Ikeda and Yamada(Ikeda and Yamada 2022) (Betz and Enerbäck(Betz and Enerbäck 2018), little is known about the particularities of how PM20D1 is regulated and how genetic variants of this gene could influence thermogenesis. PM20D1 is a secreted enzyme produced by brown and beige adipocytes. Once secreted, it catalyzes the condensation of fatty acids and amino acids to produce N-fatty acyl amino acids, which can enter the adipocytes, bind to the mitochondria, and act as endogenous uncouplers, independently of UCP1 (Long et al.(Long et al. 2016). Variations in the expression of*PM20D1*, which are due to either genetic or epigenetic mechanisms, are associated with Alzheimer’s and Parkinson’s diseases (Sanchez-Mut et al. (Sanchez-Mut et al. 2018) (Pihlstrøm et al.(Pihlstrøm et al. 2015). In addition, it has been shown that variants in the upstream region of the human*PM20D1* gene can modify methylation and transcription factor binding, which regulates the circulating levels of PM20D1; this has been proposed as a mechanism linking PM20D1 with obesity and neurodegenerative diseases (Benson et al. (Benson et al. 2019). However, except for the experimental manipulation of PM20D1 levels (Long et al.(Long et al. 2016) (Long et al.(Long et al. 2018), no study has shown that naturally occurring variations on the*PM20D1/Pm20d1* gene can impact the metabolic phenotype.

It has been recently shown that mouse strains commonly used in experimental research present distinct levels of PM20D1, which is associated with differences in the metabolic and thermogenic phenotypes (Simoes et al. (Simoes et al. 2025). BALB/c mice present greater glucose and cold tolerance than C57BL/6 and Swiss mice; this occurs independently of*Ucp1* expression but is associated with greater levels of *Pm20d1* (Simoes et al. (Simoes et al. 2025). In the present study, we identified a gain-of-function variant in the*Pm20d1* promoter region that is present in BALB/c and absent in C57BL/6 mice. In addition, using genetic approaches to either increase or decrease the levels of *Pm20d1*, we show that the metabolic and thermogenic differences between BALB/c and C57BL/6 mice can be shifted by interventions to either reduce or increase the levels of *Pm20d1*. Finally, we show that variants in the promoter region of the *PM20D1* gene in humans are associated with obesity and metabolic syndrome.

## Results

**Fig. 1:**
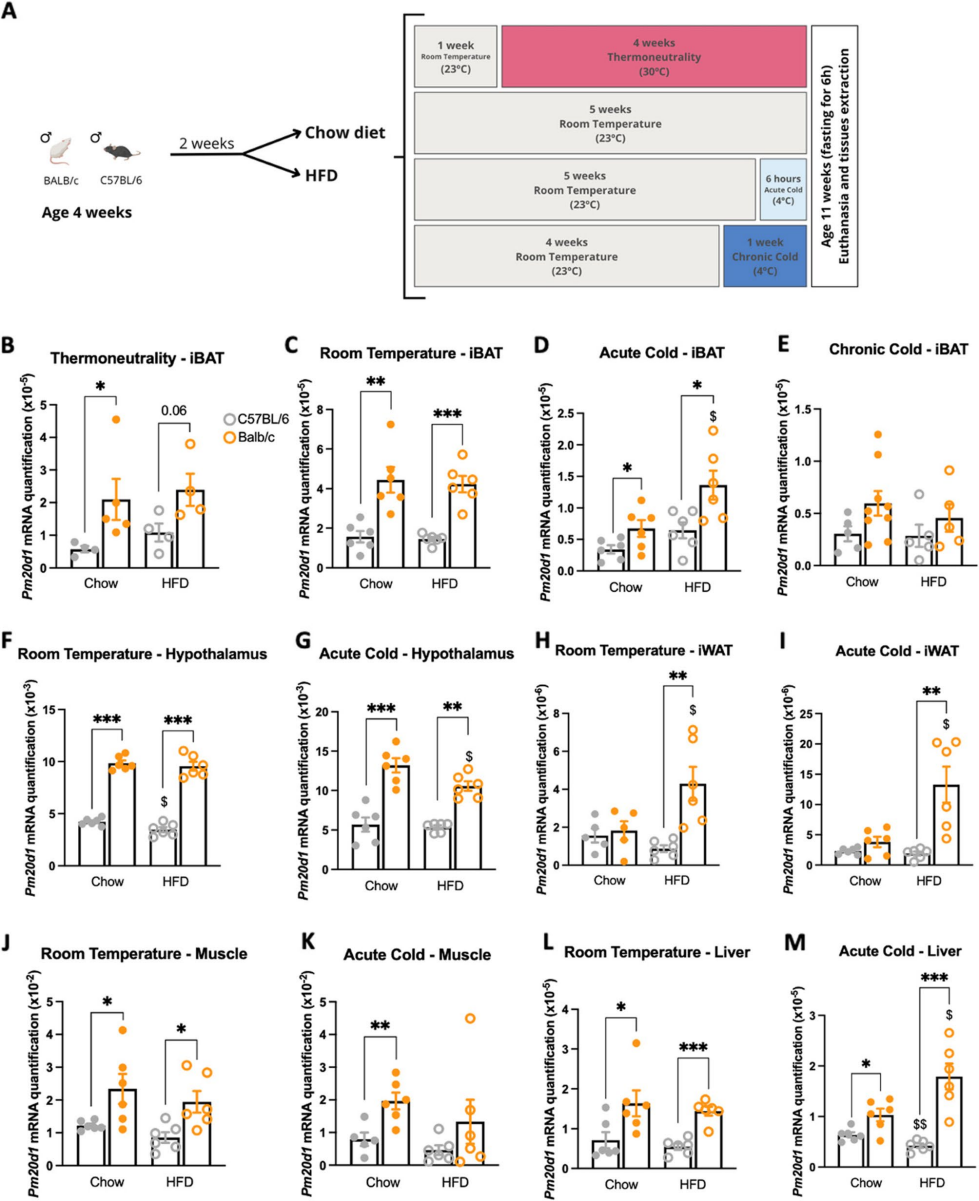
***Pm20d1*** expression across different mouse strains and tissues. **A** Experimental design; **B**-**E** Expression in the interscapular brown adipose tissue in thermoneutrality (30 °C for 4 weeks), room temperature (23 °C), acute cold (4 °C for 6-h), and chronic cold (4 °C for 1 week), respectively (*n* = 4–9); **F** and **G** Expression in the hypothalamus at room temperature and acute cold, respectively (*n* = 6); **H** and **I** Expression on the inguinal white adipose tissue in room temperature and acute cold, respectively (*n* = 5–6); **J** and **K** Expression in the gastrocnemius muscle at room temperature and acute cold, respectively (*n* = 5–6); **L** and **M** Expression in the liver at room temperature and acute cold, respectively (*n* = 6). In all: male mice at 11 weeks of age after six hours fasting; values are expressed as mean ± SEM, *p < 0.05, **p < 0.01, ***p < 0.001, independent samples Student’s T-test or Mann-Whitney test; $ *p* < 0.05, $$ *p* < 0.01, independent samples Student’s T-test or Mann-Whitney test, Chow X HFD. iBAT: interscapular brown adipose tissue; iWAT: inguinal white adipose tissue; HFD: high-fat diet

*BALB/c mice express greater levels of Pm20d1 than C57BL/6 mice in several tissues of the body.* We have recently shown that blood levels and BAT expression of Pm20d1/*Pm20d1* are greater in BALB/c as compared to Swiss and C57BL/6 mice (Simoes et al. (Simoes et al. 2025). We expanded this investigation by comparing BALB/c versus C57BL/6 fed either chow or a high-fat diet (HFD). In the BAT, the transcript levels of*Pm20d1* are greater in BALB/c under thermoneutrality (Fig. 1A and B), room temperature (Fig. 1C), and acute cold exposure (Fig. 1D). In contrast, no differences were detected when mice were chronically exposed to cold (Fig. 1E). In the hypothalamus, BALB/c mice present greater expression of *Pm20d1* either at room temperature (Fig. 1F) or acute cold exposure (Fig. 1G). In the inguinal white adipose tissue (iWAT), BALB/c mice exhibit greater expression of *Pm20d1*, both at room temperature (Fig. 1H) and following acute cold exposure (Fig. 1I), a phenomenon that is specific to those fed an HFD. In the skeletal muscle, BALB/c mice present greater expression of *Pm20d1* at room temperature (Fig. 1J); whereas under acute cold exposure, it occurs only in mice fed chow (Fig. 1K). In the liver, BALB/c present greater expression of *Pm20d1* either at room temperature (Fig. 1L) or acute cold exposure (Fig. 1M). In Supplementary Fig. 1, we compared *Pm20d1* expression at different temperatures for each mouse strain. In iBAT, for both BALB/c and C57BL/6, and fed chow or HFD, the expression of *Pm20d1* was greater at room temperature. In the muscle and liver, the expression of *Pm20d1* presented no differences between different temperatures or different strains. In the hypothalamus and iWAT, *Pm20d1* expression was greater under acute cold in both BALB/c and C57BL/6.

In Supplementary Table 1, we performed a General Linear Model (GLM) analysis to explore the interaction and effect of the three independent variables (mouse strain, temperature, and diet) in the expression of the *Pm20d1* gene in the five tissues analyzed. Across all tissues, the statistical model explained varying proportions of the differences in *Pm20d1* expression, as indicated by partial eta squared values. In BAT, the model accounted for 80% of the variance, with temperature emerging as the strongest predictor, followed by mouse strain. Both variables had significant main effects, and importantly, the interaction between mouse strain and temperature indicated that the temperature response differed between mouse strains. In iWAT, the model explained 67% of the variance, with mouse strain being the most influential factor, followed by temperature and diet. Significant interactions between mouse strain, temperature, and diet suggest that the effects of these environmental factors on *Pm20d1* expression depend on genetic background. In the hypothalamus, the model explained 88% of the variance, again with mouse strain as the dominant variable, followed by temperature and diet. For muscle and liver, the models explained 40% and 61%, respectively, of the variance, with mouse strain being the only significant predictor, indicating a consistent genetic effect independent of temperature or diet in these tissues.

**Fig. 2:**
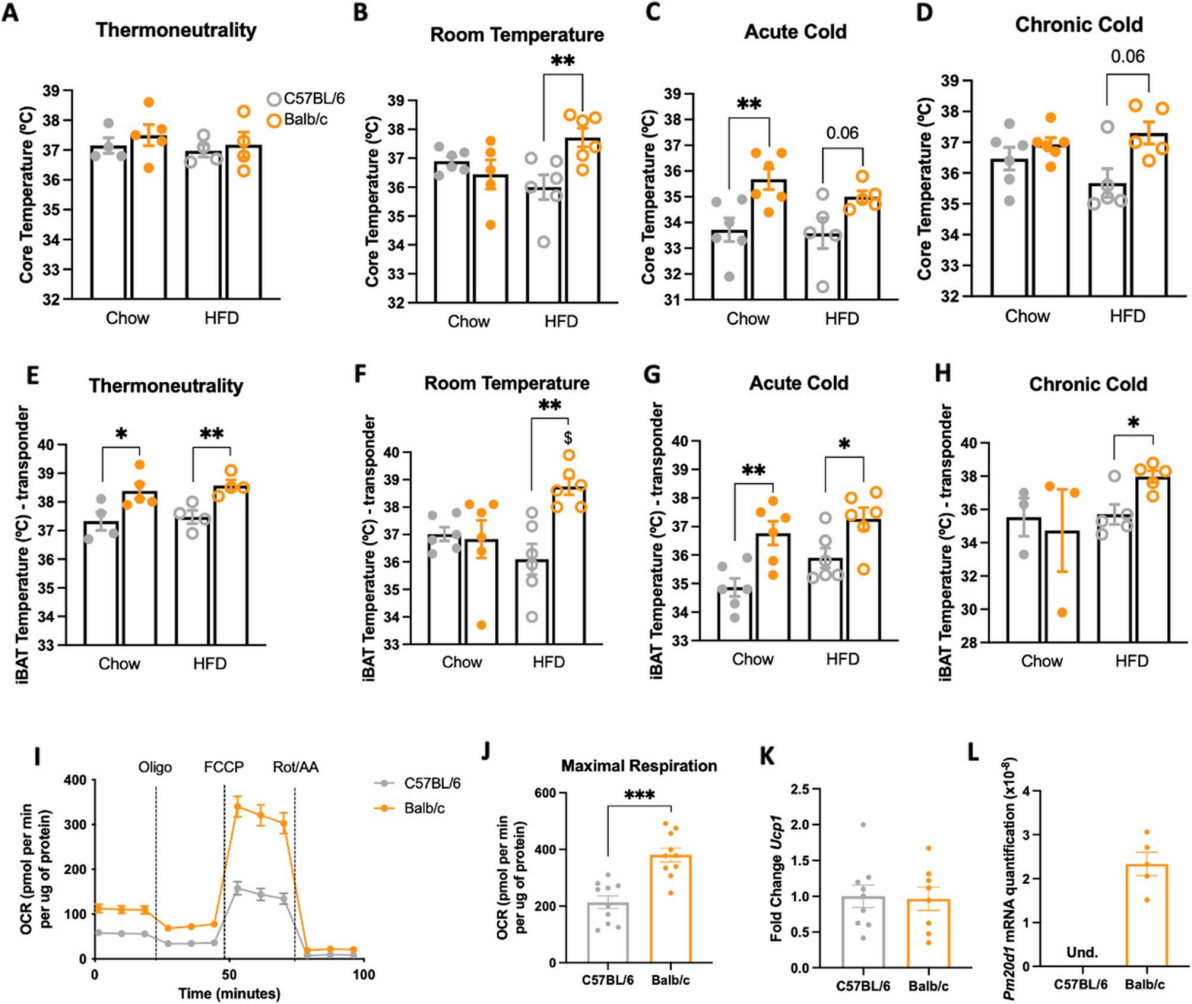
Thermogenic differences between BALB/c and C57BL/6 mice. **A**-**D** Core temperature at the end of six hours fasting in thermoneutrality, room temperature, acute cold, and chronic cold, respectively (*n* = 4–6); **E**-**H** iBAT temperature at the end of six hours fasting in thermoneutrality, room temperature, acute cold, and chronic cold, respectively (*n* = 3–6); **I** Oxygen consumption rate of primary brown adipocytes derived from C57BL/6 and BALB/c (*n* = 5 in 2 technical replicates); **J** Maximal oxygen consumption rate of primary brown adipocytes derived from C57BL/6 and BALB/c (*n* = 10); **K** and **L** *Ucp1* fold change and *Pm20d1* expression, respectively, of primary brown adipocytes derived from C57BL/6 and BALB/c.(*n* = 5–9) In A-H: male mice at 11 weeks of age. In all: values are expressed as mean ± SEM, *p < 0.05, **p < 0.01, ***p < 0.001, independent samples Student’s T-test or Mann-Whitney test; $ *p* < 0.05, independent samples Student’s T-test or Mann-Whitney test, Chow X HFD. UCP1: uncoupling protein-1; Pm20d1: peptidase M20 domain containing 1; OCR: oxygen consumption rate; Oligo: oligomycin; Rot/AA: rotenone/antimycin A; Und: undetermined

*BALB/c mice have greater thermogenic activity and greater brown adipose tissue respiration than C57BL/6 mice.* There are no differences in core temperature between BALB/c and C57BL/6 under thermoneutrality (Fig. 2A). Under room temperature, BALB/c has a higher core temperature when fed on an HFD (Fig. 2B). BALB/c has a higher core temperature when fed on chow and exposed to acute cold (Fig. 2C). In contrast, there are no differences between the strains when mice are chronically exposed to cold (Fig. 2D). The BAT temperature was higher in BALB/c under thermoneutrality (both diets) (Fig. 2E); at room temperature (under HFD) (Fig. 2F); after acute cold (both diets) (Fig. 2G); and after chronic cold exposure (under HFD) (Fig. 2H). In a previous study, we performed an Oxygen Consumption Rate (OCR) experiment, using the equipment OROBOROS (O2k Oxygraph, OROBOROS Instruments), in the iBAT of BALB/c, C57BL/6, and Swiss mice after acute cold exposure. We showed that BALB/c, on chow and HFD, had greater OCR than the two other mouse strains, with no differences in UCP1 function (Simoes et al. (Simoes et al. 2025). Next, we prepared primary brown adipocyte cultures from BALB/c and C57BL/6 mice at room temperature for respirometry experiments. As shown in Fig.2I and J, BALB/c adipocytes present greater oxygen consumption than the adipocytes from C57BL/6 at the basal state, after oligomycin addition, and, mainly after FCCP, which represents maximal respiration. There were no differences in the brown adipocytes’ expression of *Ucp1* (Fig. 2K); surprisingly, the *Pm20d1* transcripts were not detected in the brown adipocytes of C57BL/6 mice (Fig. 2L).

**Fig. 3:**
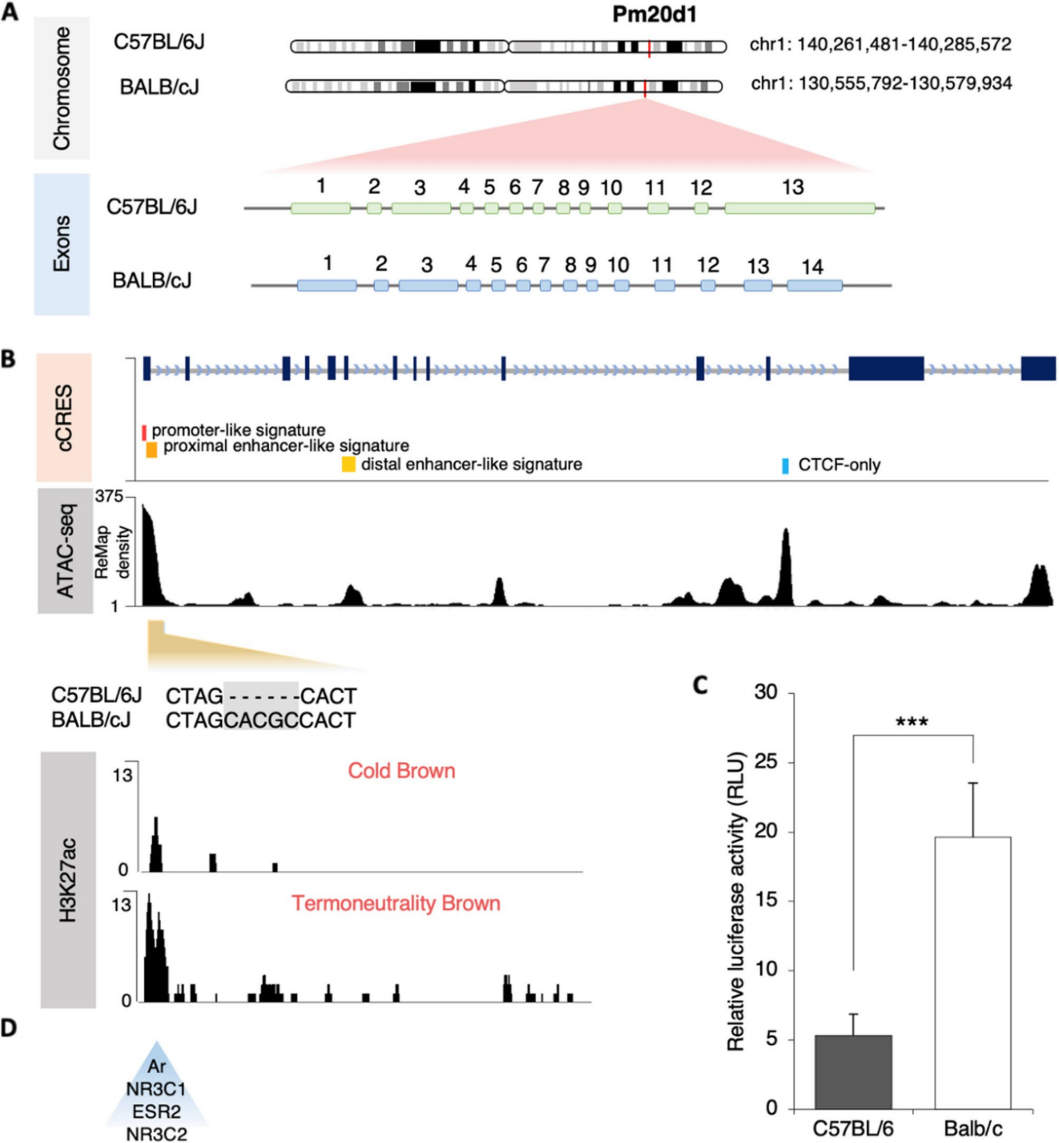
Genetic data on the animal models. **A** *Pm20d1* gene alignment of BALB/cJ and C57BL/6J; **B** cCRES and ATAC-seq signal for *Pm20d1*, and H3K27ac signal in animals’ brown adipocytes under cold exposure and thermoneutrality; **C** Transcriptional activity of the promoter region of *Pm20d1* of BALB/c and C57BL/6, shown with relative luciferase activity in 9B cells (*n* = 5 in 3 technical replicates); **D** Candidate transcription factors that could bind to the promoter region of the BALB/c mice, shown by motif analysis. In C: values are expressed as mean ± SEM, ***p < 0.001, independent samples Mann-Whitney test. cCRES: Candidate cis-Regulatory Elements; AR: androgen receptor; NR3C1: glucocorticoid receptor; ESR2: estrogen receptor 2; NR3C2: mineralocorticoid receptor

*The BALB/c mice Pm20d1 promoter region has increased transcriptional activity as compared to C57BL/6.* Because of the wide differences in *Pm20d1* expression between BALB/c and C57BL/6 mice, we hypothesized that genetic variants implicated in differential expression of the gene could exist in the strains. In C57BL/6, the *Pm20d1* gene is localized in chromosome 1:140,261,481-140285,572, whereas in BALB/c mice the *Pm20d1* gene is at chromosome 1:130,555,792 − 130,579,934 (Fig. 3A, under chromosome). The alignment of the genes revealed a structural difference, as in the C57BL/6 gene, there are 13 exons, whereas in the BALB/c there are 14 exons (Fig. 3A, exon structure). Moreover, the nucleotide alignment showed differences in two regions, promoter and exon 13, in C57BL/6; and promoter and intron between exons 13 and 14, in BALB/c (Fig. 3A, nucleotides). In Fig. 3B we depict the available data on candidate cis-regulatory elements (cCRES) across all cell types, alongside the relationship with ATAC-seq data, a specific marker for histone modifications (H3K4me3 and H3K27ac) sourced from a public database (genome.ucsc.edu). Using this approach, it was identified that within the enrichment region of the histone modifications, there are regulatory regions that differ between BALB/c and C57BL/6. To further explore the differences present in the promoter region and cCRES identified candidates, we measured their transcriptional activity using a luciferase assay. As shown in Fig. 3C, the *Pm20d1* promoter region of the BALB/c mice has greater transcriptional activity than the C57BL/6 sequence (Fig. 3C). We also performed a motif analysis to search for transcription factors that could bind to the promoter region of the BALB/c mice (chromosome 1:131.797.486-131.797.777). As shown in Fig. 3D, there are binding sites for androgen receptor, glucocorticoid receptor, estrogen receptor 2, and mineralocorticoid receptor.

**Fig. 4:**
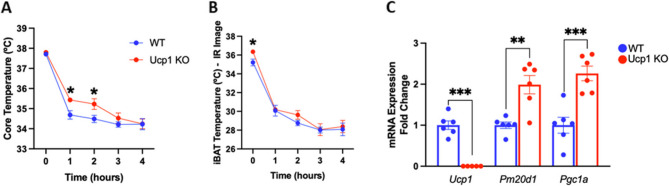
*Ucp1* knockout mice. **A** and **B** Core and iBAT temperatures, respectively, during four hours of acute cold exposure of *Ucp1* KO mice compared with wild-type (*n* = 6–7); **C** *Ucp1*,* Pm20d1*, and *Pgc1a* expression shown as fold change in *Ucp1* KO and wild-type. In all: male mice at 10–11 weeks of age, values are expressed as mean ± SEM, *p < 0.05, **p < 0.01, ***p < 0.001, independent samples Student’s T-test. KO: knockout; IR: infrared; WT: wild-type; Pgc1a: Peroxisome proliferator-activated receptor gamma coactivator 1-alpha

*Ucp1 knockout mice have greater levels of Pm20d1 transcripts than wild-type mice.* The demonstration that *Ucp1* KO mice retained thermogenic capacity and uncoupled mitochondrial respiration was key for identifying the currently known UCP1-independent mechanisms of thermogenesis (Ikeda et al. (Ikeda et al. 2017). Here, we asked if*Ucp1* KO mice express *Pm20d1* differently from wild-type mice. As shown in Fig. 4A, at baseline under room temperature, *Ucp1* KO and wild-type mice have equal core temperatures; however, after cold exposure, *Ucp1* KO mice present a higher temperature. Regarding the interscapular temperature, *Ucp1* KO mice present a higher temperature at baseline and equal temperature after cold exposure (Fig. 4B). In Fig. 4C, BAT transcripts were measured, and as expected, there were no detectable transcripts for *Ucp1*; however, both *Pm20d1* and *Pgc1a* levels were greater in *Ucp1* KO as compared to wild-type mice.

**Fig. 5:**
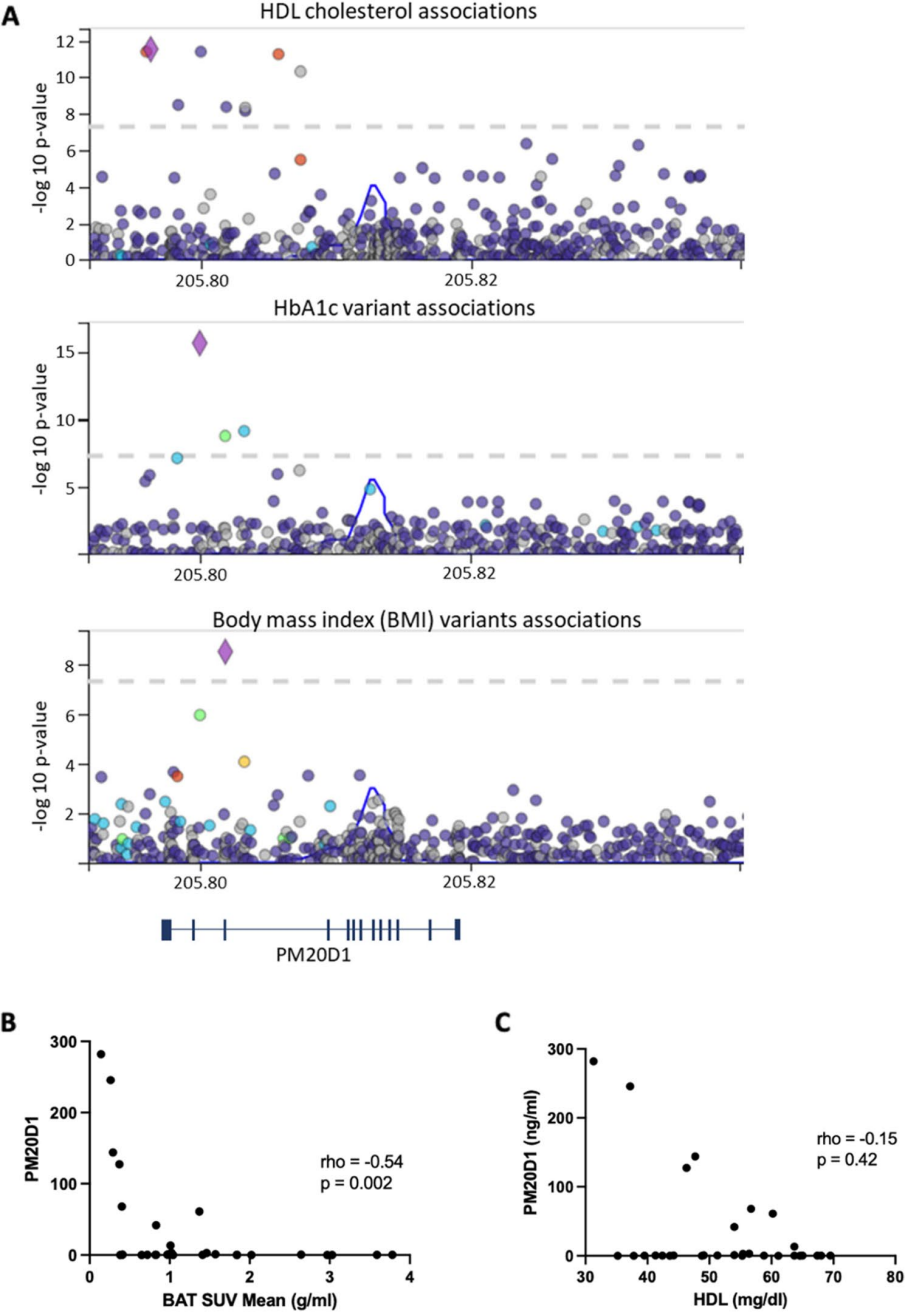
Human genetic/phenotype data. **A** LocusZoom regional association plot in the *PM20D1* gene locus. The X-axis represents chromosomal location. The Y-axis represents -log10 (*P*-value). Relation with anthropometric (body mass index), lipids (HDL cholesterol), and glycemia (HbA1c); **B** Correlation of circulating PM20D1 values and BAT SUV Mean value in human volunteers (*n* = 30); **C** Correlation of circulating PM20D1 values and HDL values in human volunteers (*n* = 30). In **B** and **C**: Spearman’s coefficients. HDL: high-density lipoprotein; HbA1c: glycated hemoglobin; BAT SUV Mean: brown adipose tissue mean standard uptake value

*Variants of the human PM20D1 gene are associated with metabolic phenotypes.* Next, we performed a genetic association analysis of the human variants of the *PM20D1* gene. As shown in Fig. 5A, there are variants associated with HDL cholesterol (upper panel), glycated hemoglobin (middle panel), and body mass index (lower panel). Most credible sets are located at the upstream region of the gene, suggesting that the initial region could be a hotspot for variants leading to the most relevant phenotypes. In addition, in human volunteers, we correlated the blood levels of PM20D1 with BAT activity (Fig. 5B) and blood HDL (Fig. 5C) distributed along a wide magnitude range. We used the BAT SUV Mean to measure BAT activity, which corresponds to the mean standard uptake value of the radiotracer (FDG18) during the PET/CT. In BAT SUV Mean, there was an inverse correlation with PM20D1.

**Fig. 6:**
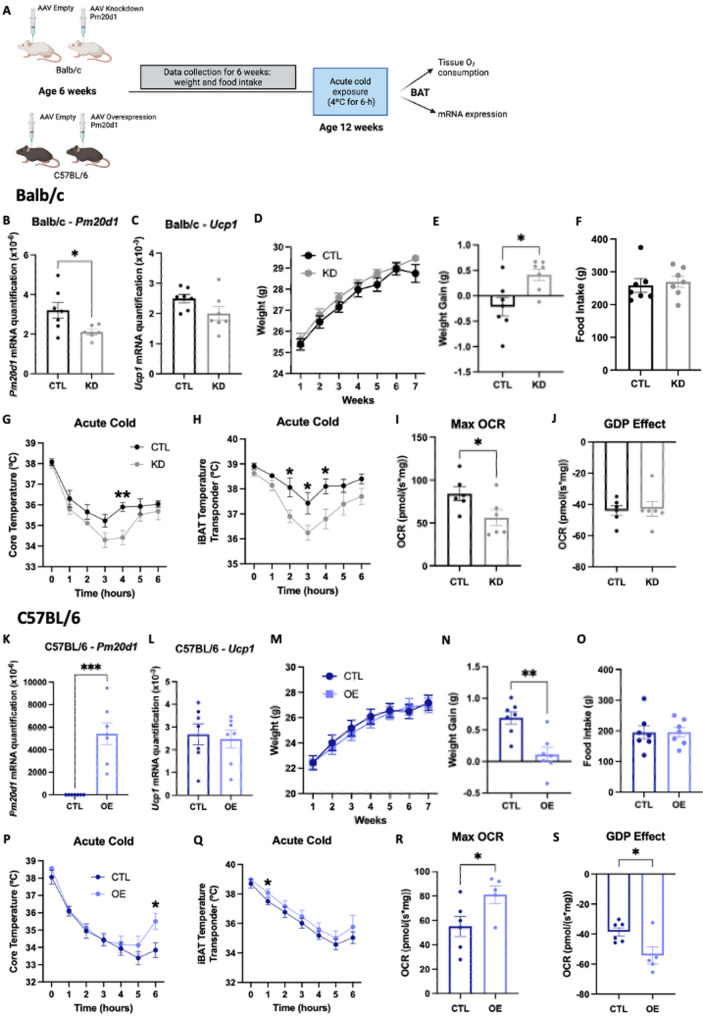
Adeno-associated viral (AAV) injection into iBAT. **A** Experimental design; **B**-**J** AAV injection to knockdown *Pm20d1* in BALB/c; **B** and **C** iBAT *Pm20d1* and *Ucp1* expression, respectively (*n* = 6–7); **D** Weight throughout the experiment, AAV injection was done right after measurement on week 1 (*n* = 7); **E** Weight gain in the last measurement (*n* = 7); **F** Total food intake (*n* = 7); **G** and **H** Core and iBAT temperatures, respectively, during six hours of acute cold exposure (*n* = 6–7); **I** iBAT maximal oxygen consumption rate (*n* = 6); **J** GDP effect on iBAT oxygen consumption rate (*n* = 6); **K**-**S** AAV injection to overexpress *Pm20d1* in C57BL/6; **K** and **L** iBAT *Pm20d1* and *Ucp1* expression, respectively (*n* = 7); **M** Weight throughout the experiment, AAV injection was done right after measurement on week 1 (*n* = 7); **N** Weight gain in the last measurement (*n* = 7); **O** Total food intake (*n* = 7); **P** and **Q** Core and iBAT temperatures, respectively, during six hours of acute cold exposure (*n* = 6–7); **R** iBAT maximal oxygen consumption rate (*n* = 5–6); **S** GDP effect on iBAT oxygen consumption rate (*n* = 5–6). In all: male mice at 12 weeks of age, values are expressed as mean ± SEM, *p < 0.05, **p < 0.01, ***p < 0.001, independent samples Student’s T-test or Mann-Whitney test. CTL: control; KD: knockdown; Max: maximal; GDP: guanosine diphosphate; OE: overexpression

*Shifts in the BAT levels of Pm20d1 promote bidirectional changes in metabolic parameters.* To explore the possibility of changing metabolic phenotypes by intervening on BAT *Pm20d1*, we created a paradigm in which, using adeno-associated viral constructs, we promoted a reduction in *Pm20d1* in BALB/c mice and an increase in *Pm20d1* in C57BL/6 mice. In BALB/c, the intervention resulted in 40% reduction in BAT *Pm20d1* (Fig. 6B), without affecting the expression of *Ucp1* (Fig. 6C). This was accompanied by a minor increase in body mass gain (Fig. 6D and E) without change in food intake (Fig. 6F), and percentage of fat mass and lean body mass (Supplementary Fig. 2 A and 2B). In addition, mice became cold-intolerant (Fig. 6G and H), and the BAT presented a reduction in tissue respiration (Fig. 6I), independently of changes in GDP-sensitive respiration, which reflects UCP1 function (Fig. 6J). There were no differences in iBAT and iWAT weight% (Supplementary Fig. 2 C and 2D). Conversely, when the opposite intervention was performed in C57BL/6, there was a significant increase in the expression of BAT *Pm20d1* (Fig. 6K) with no modification in *Ucp1* (Fig. 6L). This was accompanied by a reduction in body mass gain (Fig. 6M and N) with no change in food intake (Fig. 6O), and percentage of fat mass and lean body mass (Supplementary Fig. 2E and 2 F). Animals were also protected against cold exposure (Fig. 6P and Q), and had increased BAT respiration (Fig. 6R), in this case with some involvement of UCP1 (Fig. 6S). There were no differences in iBAT and iWAT weight% (Supplementary Fig. 2G and 2 H).

**Fig. 7:**
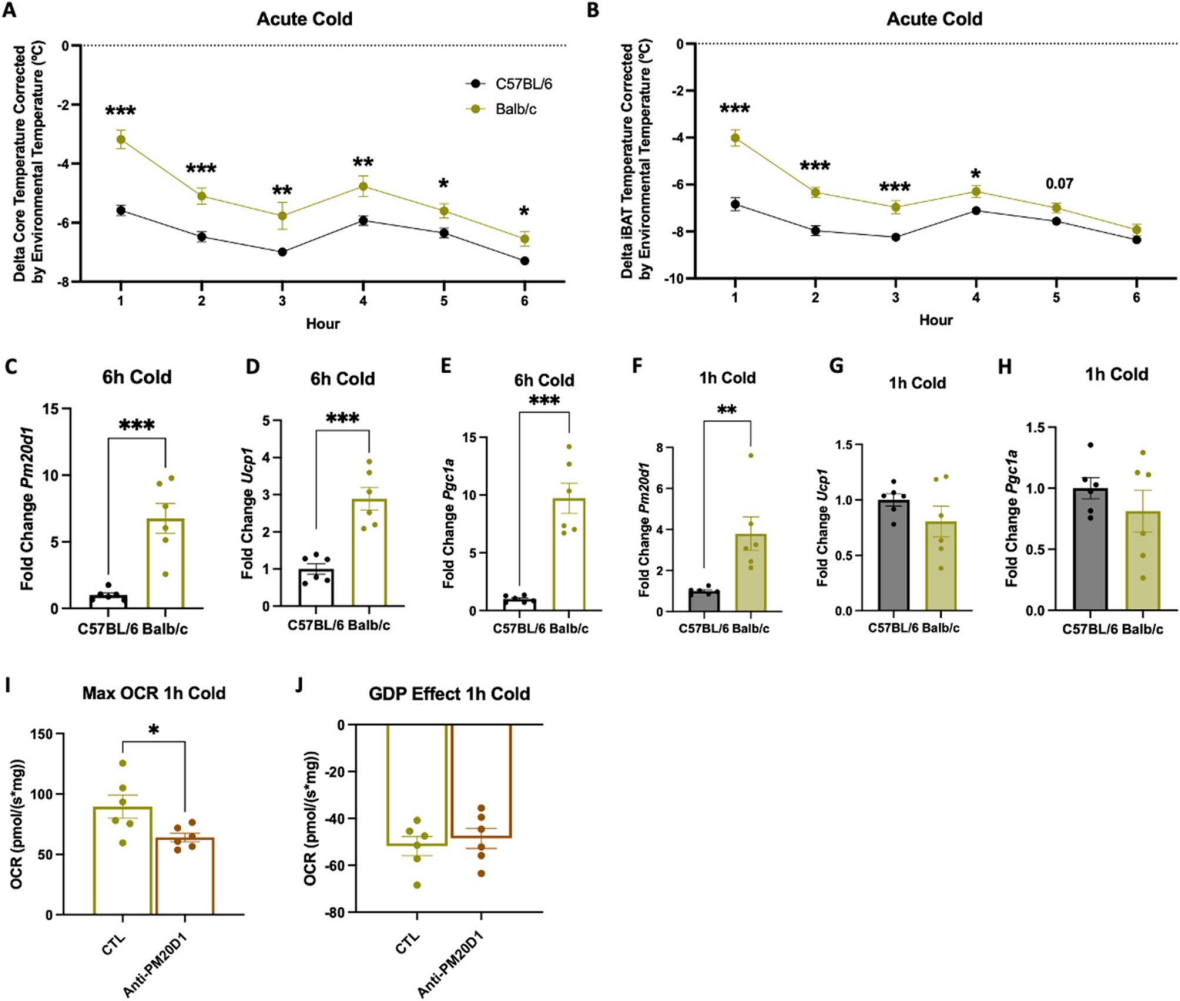
Acute cold exposure in BALB/c and C57BL/5 neonatal mice. **A** and **B** Delta core and iBAT temperatures, respectively, during six hours of acute cold exposure (*n* = 6–12); **C**-**E** iBAT *Pm20d1*,* Ucp1* and *Pgc1a* expression, respectively, after six hours of acute cold, shown as fold change (*n* = 6); **F**-**H** iBAT *Pm20d1*,* Ucp1* and *Pgc1a* expression, respectively, after one hour of acute cold, shown as fold change (*n* = 6); **I** and **J** iBAT maximal oxygen consumption rate and GDP effect on oxygen consumption rate, respectively, in BALB/c mice, at postnatal day 10, after chronic treatment with anti-PM20D1 (*n* = 6). In all: values are expressed as mean ± SEM, *p < 0.05, **p < 0.01, ***p < 0.001, independent samples Student’s T-test

*In neonatal mice*,* Pm20d1 thermogenic action precedes Ucp1 induction as a protective factor against cold intolerance.* In neonatal life, the capacity to rapidly respond to cold exposure by activating an efficient thermogenic response can represent a lifesaving adaptation. As shown in Fig. 7A and B, neonatal BALB/c mice have a more pronounced thermogenic response when exposed to cold than C57BL/6. The greater thermogenic differences occur early after starting the intervention, and gradually they fade away. Six hours after the beginning of the cold-exposure intervention (18 °C), BALB/c mice present greater expressions of *Pm20d1*, *Ucp1*, and *Pgc1a* compared to C57BL/6 mice (Fig. 7C and E). However, one hour after the beginning of the intervention, only *Pm20d1* expression is greater in BALB/c (Fig. 7F and H), suggesting that it acts as a very early thermogenic response mechanism, which precedes the canonical UCP1-dependent thermogenic response. Furthermore, in neonatal BALB/c exposed to cold for one hour, the immunoneutralization of Pm20d1 impairs cold-induced BAT respiration (Fig. 7I) independently of UCP1 (Fig. 7J).

## Discussion

The most relevant novelty of this study is the identification of a variant in the promoter region of the *Pm20d1* gene that explains, at least in part, the metabolic differences between two mouse strains commonly used in biomedical research, BALB/c and C57BL/6. This variant, present in BALB/c and absent in C57BL/6, results in a stronger promoter that leads to increased expression of *Pm20d1* in several tissues and results in protection against body mass gain and cold intolerance. Interventions that promote bidirectional changes in BAT *Pm20d1* shift the metabolic phenotypes; so, BALB/c under partial inhibition of *Pm20d1* becomes obesity prone and cold-intolerant, whereas C57BL/6 under increased expression of *Pm20d1* presents reduced body mass gain and becomes cold-tolerant. Exploring large human datasets of gene/phenotype associations, we confirmed that the upstream region of the *PM20D1* gene is a hotspot for variants associated with metabolic conditions, including body mass gain.

In 2016, Long, Spiegelman, and coworkers demonstrated that *Ucp1*-expressing cells produce and secrete PM20D1, which catalyzes the production of N-fatty acyl amino acids to act as endogenous uncouplers of mitochondrial respiration (Long et al. (Long et al. 2016). This was an important breakthrough in the field as PM20D1 joined the creatine kinase futile cycle and the sarcoplasmic/endoplasmic-reticulum calcium cycling as an additional UCP-1 independent uncoupled respiration and thermogenesis mechanism. Moreover, theoretically, as a secreted enzyme, PM20D1 and its products could be promising targets for pharmacological interventions aimed at controlling metabolic conditions.

Mechanistically, it was shown that N-fatty acyl amino acids can act upon brown adipocytes, promoting an increase of up to 300% in uncoupled respiration, which also occurs in *Ucp1* KO adipocytes (Long et al. (Long et al. 2016). This effect depends on the binding of the N-fatty acyl amino acids to SLC25 family members present in the mitochondrial membrane. Upon binding, the N-fatty acyl amino acids induce a SLC25-mediated proton flux into the mitochondrial matrix, thus resulting in a UCP1-independent mitochondrial uncoupling (Long et al.(Long et al. 2016).

The capacity of PM20D1 to increase thermogenesis and impact metabolic phenotypes has been reproduced in other studies (Benson et al. (Benson et al. 2019) (Long et al.(Long et al. 2018) (Simoes et al.(Simoes et al. 2025) (Li et al.(Li et al. 2019); however, there is at least one study reporting a negative finding regarding the capacity of N-fatty acyl amino acids to promote uncoupled respiration to a level greater than conventional fatty acids (Gao et al.(Gao et al. 2022). In that study, the authors report the testing of two pairs of compounds, each pair composed of one conventional fatty acid and one N-fatty acyl amino acid: i.e., oleate versus N-oleoyl-leucine; and arachidonate versus N-arachidonoyl-glycine. They describe that the N-acyl amino acids were competent uncouplers. Still, they did not systematically exhibit greater affinity or potency than the conventional fatty acids, and they are not as efficient uncouplers as classical protonophores (Gao et al.(Gao et al. 2022). Here, we went further in the search for evidence that PM20D1 could act independently of UCP1 to promote thermogenesis. This is evident both in the interventions in BALB/c and C57BL/6, as well as in the experiment using*Ucp1* KO mice. During cold exposure in the fed state, *Ucp1* KO animals could still maintain higher body and BAT temperatures. This was associated with elevated expression levels of *Pm20d1* and *Pgc1a* in BAT. Although some studies report that animals lacking *Ucp1* show impaired cold tolerance, several others have found the opposite, consistent with our results (Liu et al. (Liu et al. 2003). A study from the Kozak group demonstrated that*Ucp1* KO mice could adapt to cold exposure, exhibiting increased oxygen consumption and enhanced oxidation of both fat and glucose (Ukropec et al. (Ukropec et al. 2006). We acknowledge that the experiment with the*Ucp1* KO mice was not planned with the objective of providing definitive proof of the capacity of PM20D1 to act as a thermogenic factor independently of UCP1. Nevertheless, there is undisputed reproducibility regarding the impact of modifying the expression levels of PM20D1 on thermogenesis and metabolism (Long et al. (Long et al. 2016) (Long et al.(Long et al. 2018) (Simoes et al.(Simoes et al. 2025). Thus, the concept that N-acyl amino acids and PM20D1 constitute a mechanism of UCP1-independent uncouplers is not a consensus in the field and deserves further exploration.

Furthermore, we showed that BALB/c mice have higher body and iBAT temperatures compared to C57BL/6 mice, as well as increased brown adipocyte respiration. This enhanced thermogenic activity is accompanied by a greater expression of *Pm20d1* in several tissues. *Pm20d1* expression in iBAT may contribute to the elevated body temperature through adaptive thermogenesis (Celi et al. (Celi et al. 2015). In addition, BALB/c mice also exhibited increased*Pm20d1* expression in other tissues, which could further influence thermogenic activity. Both iWAT and muscle can contribute to thermogenesis, through the browning process and shivering thermogenesis, respectively (Betz and Enerbäck (Betz and Enerbäck 2018) (Wu et al.(Wu et al. 2012), and the hypothalamus plays a central role in regulating body temperature and energy expenditure; thus,*Pm20d1* may also exert thermogenic effects via the hypothalamus (Zhao et al. (Zhao et al. 2017).

Here, we did not aim at exploring the mechanism by which changes in the expression/amount of PM20D1 lead to increased thermogenesis. Instead, we aimed at defining the reason why two commonly used mouse strains present differences in the expression of *Pm20d1* and why this is associated with metabolic differences between the strains. By aligning the BALB/c and C57BL/6 genes, we provide the first evidence that a naturally occurring variant in the promoter region of the *Pm20d1* gene results in different transcriptional capacities. This is in tune with the findings of the human genome/phenotype association data showing that variants located in the promoter region of the human gene are associated with metabolic traits, such as HDL cholesterol, glycated hemoglobin, and BMI. Moreover, in the mouse genes, we identified candidate transcription factors that could bind to the BALB/c but not to the C57BL/6 promoter. Among the four transcription factors identified, ESR2 is of particular interest as it codes for the estrogen receptor 2, and estrogen is an activator of thermogenesis (Villena et al. (Villena et al. 2007) (Martínez de Morentin et al.(Martínez de Morentin et al. 2014). Early studies in the field suggested that estrogen could act directly on the BAT (Richard(Richard 1986); however, recently, studies showed that most of this action occurs through the activation of estrogen receptors in the hypothalamus, which in turn activate a sympathetic response to promote BAT thermogenesis (Martínez de Morentin et al.(Martínez de Morentin et al. 2014) (López and Tena-Sempere(López and Tena-Sempere 2016). Here, we showed that*Pm20d1* is present in the hypothalamus, and BALB/c expresses greater amounts of hypothalamic *Pm20d1* than C57BL/6. Further studies should explore the hypothesis that hypothalamic *Pm20d1* could mediate some of the effects of estrogen to promote thermogenesis in the BAT.

Another important finding of this study is the demonstration that the metabolic phenotypes of two isogenic mouse strains that naturally exhibit differences in thermogenesis and propensity to obesity can be shifted simply by modulating the expression of *Pm20d1*. In three previous studies, the levels of *Pm20d1* were changed by distinct interventions (Long et al. (Long et al. 2016) (Long et al.(Long et al. 2018) (Simoes et al.(Simoes et al. 2025); however, this has never been performed in strains that, in the baseline, express different levels of*Pm20d1*. Taken together with studies showing that humans have variants of the *PM20D1* gene that associate with metabolic phenotypes (Benson et al. (Benson et al. 2019)^24^, our results suggest that pharmacological strategies that increase PM20D1 could be an interesting intervention to treat metabolic diseases in selected patients identified by the presence of variants that impair the expression of PM20D1.

Adaptive thermogenesis is extremely important during the early days of life, as, to survive the acute change in temperature from the intra- to the extrauterine environment, the newborns must rely on an effective capacity to activate the BAT (Bienboire-Frosini et al. (Bienboire-Frosini et al. 2023). In the final part of our study, we asked if PM20D1 could be involved in newborn thermogenesis. Compared to C57BL/6, BALB/c newborns present a more efficient thermogenic response to cold. Interestingly, in this context, the induction of*Pm20d1* occurred earlier than *Ucp1*, and its immunoneutralization impaired BAT respiration independently of UCP1. Thus, PM20D1 emerges not only as a UCP1-independent mechanism of thermogenesis in adult life but also as a rapid thermogenic mechanism that precedes the activation of *Ucp1* in newborn mice.

In conclusion, the present study has expanded the understanding of how PM20D1 acts as an effector of adaptive thermogenesis. We identified a naturally occurring variant in the promoter region that explains at least in part the metabolic and thermogenic differences between two mouse strains that are commonly used in research; we demonstrate that humans also have *PM20D1* variants that are associated with metabolic phenotypes, and some of these variants are located in the promoter region; we showed that bidirectional shifts in the expression of *Pm20d1* impact the metabolic phenotypes of mice; and, we demonstrated that *Pm20d1* expression in BAT precedes the UCP1-dependent response when newborn mice are exposed to cold. While our data strongly support the role of PM20D1 in metabolic regulation, further studies are required to elucidate the precise molecular mechanisms involved in these actions. Nonetheless, together with prior studies, our data reinforce the potential that PM20D1 represents as a candidate for therapeutic intervention in metabolic disorders.

## Materials and methods

### Mice models

Male BALB/c (Balb/c) and C57BL/6J (C57BL/6) mice, four weeks old, were obtained from the Multidisciplinary Center for Biological Investigation on Laboratory Animal Science at the University of Campinas (Campinas, São Paulo, Brazil). Mice were housed individually at room temperature (23 °C) with a 12:12 h light/dark cycle and *ad libitum* access to water and food. When mice were six weeks old, they were divided into two groups, using Z-randomization according to their weight. Therefore, each mouse strain received either a standard chow diet or a high-fat diet (HFD) containing 45% of the calories from fats (Table 1). Animals from four different cohorts were maintained at room temperature (23 °C), acute cold exposure (4 °C), chronic cold exposure (4 °C), or thermoneutrality (30 °C). Animals at 11 weeks old were submitted to 6 h of fasting and euthanasia (Fig. 1A). All experiments with mice were performed in agreement with and approved by the Institutional Animal Care and Use Committee (protocol number: 6139-1/2022).

**Table 1:** Composition of the diets

	High-Fat Diet (HFD − 45%)			Standard Diet (Chow)		
Ingredients	g	kcal	%kcal	g	kcal	%kcal
***Total fat***	0.268	2.412	48	0.045	0.41	10
*Soy Oil*	0.04			0.045		
*Animal Lard*	0.228					
***Total carbohydrate***	0.492	1.97	39	0.68	2.72	70
*Sucrose*	0.1					
*Dextrin*	0.132					
*Corn Starch*	0.1995			0.55		
*Fiber*	0.05			0.08		
***Total protein***	0.2	0.68	13	0.225	0.77	20
*Casein*	0.2			0.225		
***Mix minerals***	0.035			0.05		
***Mix vitamins***	0.010			0.05		
***L-cysteine***	0.003					
***Choline bitartrate***	0.0025					
***Total***	1	5.06		1	3.89	

### Adeno-associated viral injection

Viral vector was injected, using an ultra-fine insulin syringe, directly into the intrascapular brown adipose tissue (iBAT) (30 µl), in multiple locations, of BALB/c and C57BL/6 male mice when they were six weeks old. BALB/c mice received shRNA scramble as a control and AAV (mPm20d1[shRNA#1]) to decrease (knockdown) *Pm20d1*. C57BL/6 mice received GFP as a control and AAV (CAG > mPm20d1) to overexpress *Pm20d1*. The viral concentration for BALB/c was 1.2 × 10^11^ GC/ml and for C57BL/6 was 6.5 × 10^10^ GC/ml (Bates et al. (Bates et al. 2020). Mice were kept on a standard chow diet for six weeks. During this period, body weight and food intake were measured every week, and at 10 weeks of age, body composition was analyzed by magnetic resonance imaging (Bruker; Minispec LF50). When animals reached 12 weeks, they were exposed to acute cold and were submitted to euthanasia for blood and tissue collection (Fig.6A). A fragment of iBAT was obtained and immediately placed on a conservation buffer (BIOPS) for tissue oxygen consumption experiment (Santos et al. (Santos et al. 2025); the remaining of the tissues were stored at−80 °C for further analysis.

### Acute cold exposure

Animals were exposed to 6 h of acute cold (4 °C) (de-Lima-Júnior et al. (de-Lima-Júnior et al. 2019). Mice were maintained individually in a fasting state throughout the experiment. During this period, we analyzed core temperature, using a rectal probe (BIOSEB; BIOTK8851), and iBAT temperature using an implantable temperature transponder (Avidity Science; BMDS; HTEC IPTT-300) every hour (0, 1, 2, 3, 4, 5, and 6 h). After the exposure, mice were submitted to euthanasia for blood and tissue collection. The blood samples were centrifuged for 22 min at 2,500 g at 4 °C, and the serum was collected. The serum and tissues were stored at−80 °C for further analysis.

### Chronic cold exposure

Mice were exposed to chronic cold (4 °C) for one week (Shamsi et al. (Shamsi et al. 2023). Mice were housed individually with a 12:12 h light/dark cycle and*ad libitum* access to water and food. On the last day of chronic cold exposure, animals were fasted for six hours before we analyzed core and iBAT temperatures. After the fasting period, mice were submitted to euthanasia for blood and tissue collection. The blood samples were centrifuged for 22 min at 2,500 g at 4 °C, and the serum was collected. The serum and tissues were stored at −80 °C for further analysis.

### Thermoneutrality

Mice were exposed to thermoneutrality for four weeks (30 °C) (Park et al. (Park et al. 2023). Mice were housed individually with a 12:12 h light/dark cycle and*ad libitum* access to water and food. On the last day of thermoneutrality, animals fasted for six hours before we analyzed core and iBAT temperatures. After the fasting period, mice were submitted to euthanasia for blood and tissue collection. The blood samples were centrifuged for 22 min at 2,500 g at 4 °C, and the serum was collected. The serum and tissues were stored at −80 °C for further analysis.

### Ucp1 KO mice

Male, C57BL/6 and *Ucp1* KO mice, four weeks old, were maintained at room temperature and on a standard chow diet for six weeks. When animals reached 10 weeks, they were exposed to four hours of acute cold (4 °C) for five consecutive days, in a fed state. During the cold exposure, we analyzed core temperature and iBAT temperature using an infrared thermal camera (FLIR, T540sc) every hour (0, 1, 2, 3, and 4 h). At the end of the last day of exposure, when mice were 11 weeks old, they were submitted to euthanasia for blood and tissue collection serum and tissues were stored at −80 °C for further analysis. This experiment was part of another project, approved by the Institutional Animal Care and Use Committee (protocol number: 6163-1/2022).

### Neonatal experiments

BALB/c and C57BL/6 mice at postnatal day 8 (P8) were exposed to either 1–6 h of acute cold (18 °C). Mice were maintained in individual boxes in a fasting state throughout the experiment. During this period, we analyzed core temperature, measured orally, using a rectal probe (BIOSEB; BIOTK8851), and iBAT temperature using an infrared thermal camera (FLIR, T540sc) every hour (0, 1, 2, 3, 4, 5, and 6 h). Newborns, until P19, have their body temperatures oscillating according to the environmental temperature (Lagerspetz (Lagerspetz 1962). Therefore, body and iBAT temperatures were corrected by environmental temperature. We measured body weight before and after the acute cold exposure. After the exposure, we measured blood glucose using a glucose meter, and mice were submitted to euthanasia for blood and tissue collection. The serum and tissues were stored at−80 °C for further analysis.

### Anti-PM20D1 treatment

BALB/c newborn mice were treated with either Immunoglobulin G (ThermoFisher Scientific, cat. # 31235) or anti-PM20D1 (St John’s Laboratory, cat. # STJ195978) at postnatal day 6 (P6) and postnatal day 9 (P9) (0.45 µg/g/dose). The protocol was adapted to newborns from a prior study of the group (Simoes et al. (Simoes et al. 2025). At postnatal day 10, mice were exposed to 1 h of acute cold (18 °C). After the exposure, mice were submitted to euthanasia, and iBAT was extracted for the tissue oxygen consumption experiment.

### iBAT oxygen consumption

 After the acute cold exposure, a small piece of iBAT was placed into the preservation solution, BIOPS (2.77 mM of CaK2EGTA, 7.23 mM K2EGTA, 0.1 µM free calcium, 20 mM imidazole, 50 mM K +/MES, 0.5 mM dithiothreitol, 7 mM MgCl2, 5 mM ATP, and 15 mM phosphocreatine, pH 7.1) (Santos et al. (Santos et al. 2025). Two mg of the tissue was added to 1 ml of the mitochondrial respiration medium, MiR05 (10 mM KH2PO4, 3 mM MgCl2, 500 µM EGTA, 60 mM lactobionic acid, 20 mM taurine, 110 mM sucrose, 1 g/l BSA, and 20 mM HEPES, pH 7.1). Tissue was cut and placed into the respiratory chamber, with 2 ml of MiR05, kept at 37 °C (O2k Oxygraph, OROBOROS Instruments). Before the beginning of the analysis, 2 µM of digitonin was added. To stimulate respiration, pyruvate (5 mM), malate (2 mM), glutamate (10 mM), and succinate (10 mM) were sequentially titrated into the O2k chamber. The maximal oxygen consumption rate was considered the highest iBAT oxygen consumption rate under these conditions. To inhibit UCP1 activity, 4 mM of guanosine diphosphate (GDP) was titrated (Cantó and Garcia-Roves(Cantó and Garcia-Roves 2015) (Porter et al.(Porter et al. 2016).

### Primary cell culture

Adipose tissues were isolated from male C57BL/6 and BALB/c mice (5–6 weeks old) immediately after sacrifice. iBAT was dissected under sterile conditions and carefully cleaned. Tissue fragments were incubated in digestion medium composed of collagenase D (1.5 U/mL, Roche), dispase II (2.4 U/mL, Roche), and freshly added CaCl_2_ (10 mM) in 1x PBS. The suspension was mixed thoroughly and incubated at 37 °C for 20–30 min with gentle agitation (150 rpm), and digestion was monitored every 10–15 min to ensure optimal tissue dissociation without compromising cell viability. Digestion was stopped by adding complete medium DMEM/F12 (Termo Fisher Scientific) supplemented with 10% fetal bovine serum and 1% penicillin-streptomycin (Termo Fisher Scientific), and the suspension was filtered through a 70 μm cell strainer to remove undigested material. The filtrate was centrifuged at 700 × g for 10 min to separate mature adipocytes (floating layer) from the stromal vascular fraction (SVF, pellet). The adipocyte layer was discarded, and the SVF was washed twice with 1X PBS, centrifuged again, and resuspended in complete medium. Cells were seeded in 6-well plates and incubated at 37 °C with 5% CO_2_. After 3 h, the medium was replaced to remove non-adherent cells. SVF cells were expanded until ~ 80% confluence and then induced to differentiate into adipocytes using a standard adipogenic cocktail containing T3 (1 nM), IBMX (0.5 mM), dexamethasone (2 µg/mL), insulin (20 nM), rosiglitazone (1 µg/ml), and indomethacin (125 µM). Cells were then used to measure oxygen consumption rate and gene expression.

### Cell oxygen consumption rate

Oxygen consumption rate (OCR) was measured using the Seahorse XF Cell Mito Stress Test (Agilent Technologies) as previously described (Chaurasia et al. (Chaurasia et al. 2021). Briefly, primary brown adipocytes (derived from C57BL/6 and BALB/c) were seeded on day 6 of differentiation directly into Seahorse XF24 cell culture microplates. On the day of the assay, the culture medium was replaced with Seahorse XF assay medium—non-buffered DMEM (Agilent) supplemented with 10 mM glucose, 1 mM pyruvate, and 2 mM glutamine—and cells were incubated at 37 °C in a non-CO_2_ incubator for 1 h to allow temperature and pH equilibration. Mitochondrial function was assessed by sequential injection of oligomycin (5 µM), FCCP (5 µM), and a mixture of rotenone and antimycin A (1 µM and 2 µM, respectively), using a Seahorse XFe24 Analyzer (Agilent). OCR was recorded at baseline and following each injection. Values were normalized by total protein content per well, determined using the BCA Protein Assay Kit (Pierce). All conditions were measured in five technical replicates and repeated in two independent experiments.

### RNA extraction and quantitative real-time qPCR

RNA extraction was performed on iBAT, inguinal white adipose tissue (iWAT), hypothalamus, muscle (gastrocnemius), liver, and brown adipocytes. In iBAT, RNA extraction was performed using an RNA extraction kit (Life Technologies, PureLink RNA Mini Kit). In the other tissues and brown adipocytes, RNA extraction was performed using the TRIzol reagent method (Invitrogen Corporation, CA, USA). A high-capacity cDNA Reverse Transcription kit (Applied Biosystems) was used for to synthesize complementary DNA (cDNA). Gene expression analysis was performed via RT qPCR using TaqMan Universal PCR Master Mix (7500 detection system, Applied Biosystems). *18s* was employed as a reference gene for iBAT, liver, and brown adipocytes; *Actb* was employed as a reference gene for hypothalamus and muscle. For iWAT the average of *18s* and *Gapdh* was employed as the reference gene. The complete list of primers is shown in Table 2. When more than two groups were analyzed, values were presented as raw data. When only two groups were being analyzed, values were presented as fold change, using C57BL/6 as the control, unless otherwise stated.

**Table 2:** Primers used for RT qPCR

Gene Name	Assay	Ref Seq	Exon Boundary
*18s*	4319413E	X03205.1	1
*Actb*	Mm04394036_g1	AK075973.1	1–2
*Gapdh*	Mm99999915_g1	Nm_008084.2	2–3
*Pgc1α*	Mm.PT.58.16192665	NR_027710(1)	5-7a
*Pm20d1*	Mm.PT.58.9852506	NM_178079(1)	2–3
*Ucp1*	Mm.PT.58.8720419	NM_011701(1)	1–3

### Pm20d1 gene alignment and regulatory gene analysis

Comparative genomic analyses between C57BL/6J and BALB/c mice were performed using the UCSC Genome Browser (http://genome.ucsc.edu). The chromosomes 1 of BALB/cJ and C57BL/6J were retrieved from BioProject accession number PRJNA310854 using the Linux SRA Toolkit. The chromosome was aligned using the Blastn tool. To identify the region of chromosome of *Pm20d1*, we obtained annotations from the Ensembl genome browser (www.ensembl.org/) and subseted the part common to the two mice. For a detailed analysis of the region BALB/cJ (chr1: 140,261,481 − 140,285,572) and C57BL/6J (chr1: 130,555,792 − 130,579,934), we considered the ENCODE Candidate cis-Regulatory Elements combined from all cell types. Specific loci of interest were manually inspected for strain-specific sequence variations, including promoter and enhancer regions. To assess chromatin context and regulatory landscape, publicly available epigenomic datasets from the ENCODE Project were integrated. These included tracks for ATAC-seq. Tracks were overlaid and visualized using the Integrative Genomics Viewer (version 2.12.3) custom track function to compare epigenetic profiles at regions. DNA sequence conservation across mice was evaluated using MEGA 11 software (Tamura et al. (Tamura et al. 2021). For H3K27ac (GSE108077) of brown adipose tissue (Roh et al.(Roh et al. 2018), reads were aligned by using Bowtie2 (Langmead and Salzberg(Langmead and Salzberg 2012). SAM files were then converted into BAM files. Next, peak calling was determined by using MACS2 (Zhang et al.(Zhang et al. 2008). Wig to bigWig files were generated, and then peaks were visualized by the UCSC Genome Browser. We performed motif analysis to search for transcription factors in the promoter region that did not match between the two animals (chr1:131,797,486 − 131,797,777) using the JASPAR tool. Figures were generated using IGV tools or BioRender.

### Luciferase assay

The sequences inserted were the promoter region with the changes identified in the analysis: BALB/c (CTAGCACGCCACT) and C57BL/6J (CTAG-----CACT). Plasmid constructions followed a previous publication on plasmid construction (Kim et al. (Kim et al. 1998). Preadipocytes (9B cells) expressing the promoter of BALB/c, C57BL/6J, and control (without promoter) were differentiated. At day 7 of differentiation, luciferase activity was measured using the Dual-Luciferase Reporter Assay System (Promega) following the manufacturer’s instructions. Cells were washed with 1X PBS and lysed in passive lysis buffer (Promega) for 15 min at room temperature. Lysates were transferred to white 96-well plates, and both firefly and Renilla luciferase activities were sequentially measured using a luminometer with an integration time of 5 s per well. Firefly luciferase values were normalized to Renilla luciferase to account for variations in transfection efficiency and cell number. Data were expressed as relative luciferase activity (Firefly/Renilla). All conditions were measured in five technical replicates and repeated in three independent experiments.

### Human database analyses

Genetic association analyses were performed using publicly available data from the Common Metabolic Diseases Knowledge Portal (hugeamp.org), which aggregates large-scale human genetic datasets. We focused on the *PM20D1* gene locus to investigate the association of genetic variants with metabolic traits, including body mass index (BMI), HDL (high-density lipoprotein) levels, and glycated hemoglobin (HbA1c). Regional association plots were generated to visualize variant-level significance. Locus-specific analyses were restricted to common variants of the *PM20D1* gene.

### Serum PM20D1 quantification

To quantify PM20D1 in human volunteers, an enzyme-linked immunosorbent assay (ELISA) kit was used according to the manufacturer’s recommendations (MyBioSource, cat. MBS280518). For this analysis, serum from another project of our group was used (NCT03024359). Information about the data collection can be found in detail in the published article (Monfort-Pires et al. (Monfort-Pires et al. 2021).

### Statistical analysis

Values were expressed as means ± standard error of the mean (SEM), using GraphPad Prism v.8.4.3. Outliers were identified using the ROUT test, and the Shapiro-Wilk test was used to test for normal distribution. A Two-tailed independent samples Student’s t-test was used to compare two groups, and a One-Way ANOVA was used to compare three groups. A nonparametric test was used when variables did not follow a normal distribution. Correlations were tested by Spearman’s coefficients, since the PM20D1 variable is nonparametric. A general linear model (GLM) was employed with *Pm20d1* expression as the dependent variable and mouse strain, temperature, and diet as fixed factors. The model tested main effects, all two-way and three-way interactions, and the overall contribution of each factor to variance in *Pm20d1* expression across different tissues. In all analyses, *p* < 0.05 was defined as statistically significant.

## Supplementary Information

Supplementary Material 1. Supplementary Table 1. GLM summary of main effects and interactions of mouse strain, temperature, and diet on *Pm20d1* expression in brown adipose tissue, inguinal white adipose tissue, hypothalamus, muscle, and liver. Supplementary Figure 1. *Pm20d1* expression at different temperatures in BALB/c and C57BL/6, across various tissues.Expression in the interscapular brown adipose tissue in BALB/c and C57BL/6, respectively;Expression in the hypothalamus in BALB/c and C57BL/6, respectively;Expression in the inguinal white adipose tissue in BALB/c and C57BL/6, respectively;Expression in the gastrocnemius muscle in BALB/c and C57BL/6, respectively;Expression in the liver in BALB/c and C57BL/6, respectively. In all: male mice at 11 weeks of age after six hours fasting; values are expressed as mean ± SEM, *p<0.05, **p<0.01, independent samples Student’s T-test or Mann-Whitney test, and One-Way ANOVA or Kruskal-Wallis test. iBAT: interscapular brown adipose tissue; iWAT: inguinal white adipose tissue; HFD: high-fat diet. Supplementary Figure 2. Effect of Adeno-associated viralinjection into iBAT on body composition and adipose tissues.AAV injection to knockdown *Pm20d1* in BALB/c;Fat mass percentage and lean body mass percentage, respectively;iBAT weight percentage and iWAT weight percentage, respectively;AAV injection to knockdown *Pm20d1* in C57BL/6;Fat mass percentage and lean body mass percentage, respectively;iBAT weight percentage and iWAT weight percentage, respectively. In A, B, E, and F: male mice at 10 weeks of age; In C, D, G, and H: male mice at 12 weeks of age. In all: values are expressed as mean ± SEM, independent samples Student’s T-test or Mann-Whitney test. CTL: control; KD: knockdown; OE: overexpression.

## Data Availability

Data will be available upon request directed to the corresponding author, LAV (lavellos@unicamp.br).
