# Supplementary material for: Bidirectional shifts in Pm20d1 expression impact thermogenesis and metabolism
Source: Mol Med. 2025 Aug 28;31:283. doi: 10.1186/s10020-025-01345-9 (PMC12395774; doi:10.1186/s10020-025-01345-9)
Supplement: Supplementary file 1 — Supplementary Material 1. Supplementary Table 1. GLM summary of main effects and interactions of mouse strain, temperature, and diet on Pm20d1 expression in brown adipose tissue, inguinal white adipose tissue, hypothalamus, muscle, and liver. Supplementary Figure 1. Pm20d1 expression at different temperatures in BALB/c and C57BL/6, across various tissues.Expression in the interscapular brown adipose tissue in BALB/c and C57BL/6, respectively;Expression in the hypothalamus in BALB/c and C57BL/6, respectively;Expression in the inguinal white adipose tissue in BALB/c and C57BL/6, respectively;Expression in the gastrocnemius muscle in BALB/c and C57BL/6, respectively;Expression in the liver in BALB/c and C57BL/6, respectively. In all: male mice at 11 weeks of age after six hours fasting; values are expressed as mean ± SEM, *p<0.05, **p<0.01, independent samples Student’s T-test or Mann-Whitney test, and One-Way ANOVA or Kruskal-Wallis test. iBAT: interscapular brown adipose tissue; iWAT: inguinal white adipose tissue; HFD: high-fat diet. Supplementary Figure 2. Effect of Adeno-associated viralinjection into iBAT on body composition and adipose tissues.AAV injection to knockdown Pm20d1 in BALB/c;Fat mass percentage and lean body mass percentage, respectively;iBAT weight percentage and iWAT weight percentage, respectively;AAV injection to knockdown Pm20d1 in C57BL/6;Fat mass percentage and lean body mass percentage, respectively;iBAT weight percentage and iWAT weight percentage, respectively. In A, B, E, and F: male mice at 10 weeks of age; In C, D, G, and H: male mice at 12 weeks of age. In all: values are expressed as mean ± SEM, independent samples Student’s T-test or Mann-Whitney test. CTL: control; KD: knockdown; OE: overexpression. [file 10020_2025_1345_MOESM1_ESM.docx]

**Supplementary Material**

**Supplementary Table 1.** GLM summary of main effects and interactions of mouse strain, temperature, and diet on *Pm20d1* expression in brown adipose tissue, inguinal white adipose tissue, hypothalamus, muscle, and liver (GLM univariate).

| **Tissue** | **Effect** | **df** | **F** | **p-value** | **Partial η²** |
| --- | --- | --- | --- | --- | --- |
| **Brown Adipose Tissue** | Corrected Model | 15 | 19.74 | **<0.001** | **0.802** |
|  | Mouse Strain | 1 | 66.32 | **<0.001** | **0.476** |
|  | Temperature | 3 | 58.25 | **<0.001** | **0.705** |
|  | Diet | 1 | 1.14 | 0.289 | 0.015 |
|  | Mouse Strain * Temperature | 3 | 15.91 | **<0.001** | **0.395** |
|  | Mouse Strain * Diet | 1 | 0.01 | 0.967 | 0 |
|  | Temperature * Diet | 3 | 1.24 | 0.302 | 0.048 |
|  | Mouse Strain * Temperature * Diet | 3 | 0.2 | 0.895 | 0.008 |
| **Inguinal White Adipose Tissue** | Corrected Model | 7 | 11.15 | **<0.001** | **0.673** |
|  | Mouse Strain | 1 | 22.8 | **<0.001** | **0.375** |
|  | Temperature | 1 | 13.5 | **<0.001** | **0.262** |
|  | Diet | 1 | 9.75 | **0.003** | **0.204** |
|  | Mouse Strain * Temperature | 1 | 7.04 | **0.012** | **0.156** |
|  | Mouse Strain * Diet | 1 | 14.12 | **<0.001** | **0.271** |
|  | Temperature * Diet | 1 | 4.4 | **0.043** | **0.104** |
|  | Mouse Strain * Temperature * Diet | 1 | 3.74 | 0.061 | 0.09 |
| **Hypothalamus** | Corrected Model | 7 | 41.01 | **<0.001** | **0.878** |
|  | Mouse Strain | 1 | 249.15 | **<0.001** | **0.862** |
|  | Temperature | 1 | 24.74 | **<0.001** | **0.382** |
|  | Diet | 1 | 6.68 | **0.014** | **0.143** |
|  | Mouse Strain * Temperature | 1 | 0.44 | 0.511 | 0.011 |
|  | Mouse Strain * Diet | 1 | 1.352 | 0.252 | 0.033 |
|  | Temperature * Diet | 1 | 1.581 | 0.216 | 0.038 |
|  | Mouse Strain * Temperature * Diet | 1 | 3.163 | 0.083 | 0.073 |
| **Muscle** | Corrected Model | 7 | 3.66 | **0.004** | **0.396** |
|  | Mouse Strain | 1 | 18.69 | **<0.001** | **0.324** |
|  | Temperature | 1 | 3.41 | 0.072 | 0.08 |
|  | Diet | 1 | 3.08 | 0.087 | 0.073 |
|  | Mouse Strain * Temperature | 1 | 0.03 | 0.862 | 0.001 |
|  | Mouse Strain * Diet | 1 | 0.12 | 0.735 | 0.003 |
|  | Temperature * Diet | 1 | 0.05 | 0.833 | 0.001 |
|  | Mouse Strain * Temperature * Diet | 1 | 0.08 | 0.784 | 0.002 |
| **Liver** | Corrected Model | 7 | 8.98 | **<0.001** | **0.611** |
|  | Mouse Strain | 1 | 51.02 | **<0.001** | **0.561** |
|  | Temperature | 1 | 0.83 | 0.368 | 0.02 |
|  | Diet | 1 | 0.14 | 0.71 | 0.004 |
|  | Mouse Strain * Temperature | 1 | 0.01 | 0.918 | 0 |
|  | Mouse Strain * Diet | 1 | 3.54 | 0.067 | 0.081 |
|  | Temperature * Diet | 1 | 3.25 | 0.079 | 0.075 |
|  | Mouse Strain * Temperature * Diet | 1 | 4.05 | **0.051** | 0.092 |

**
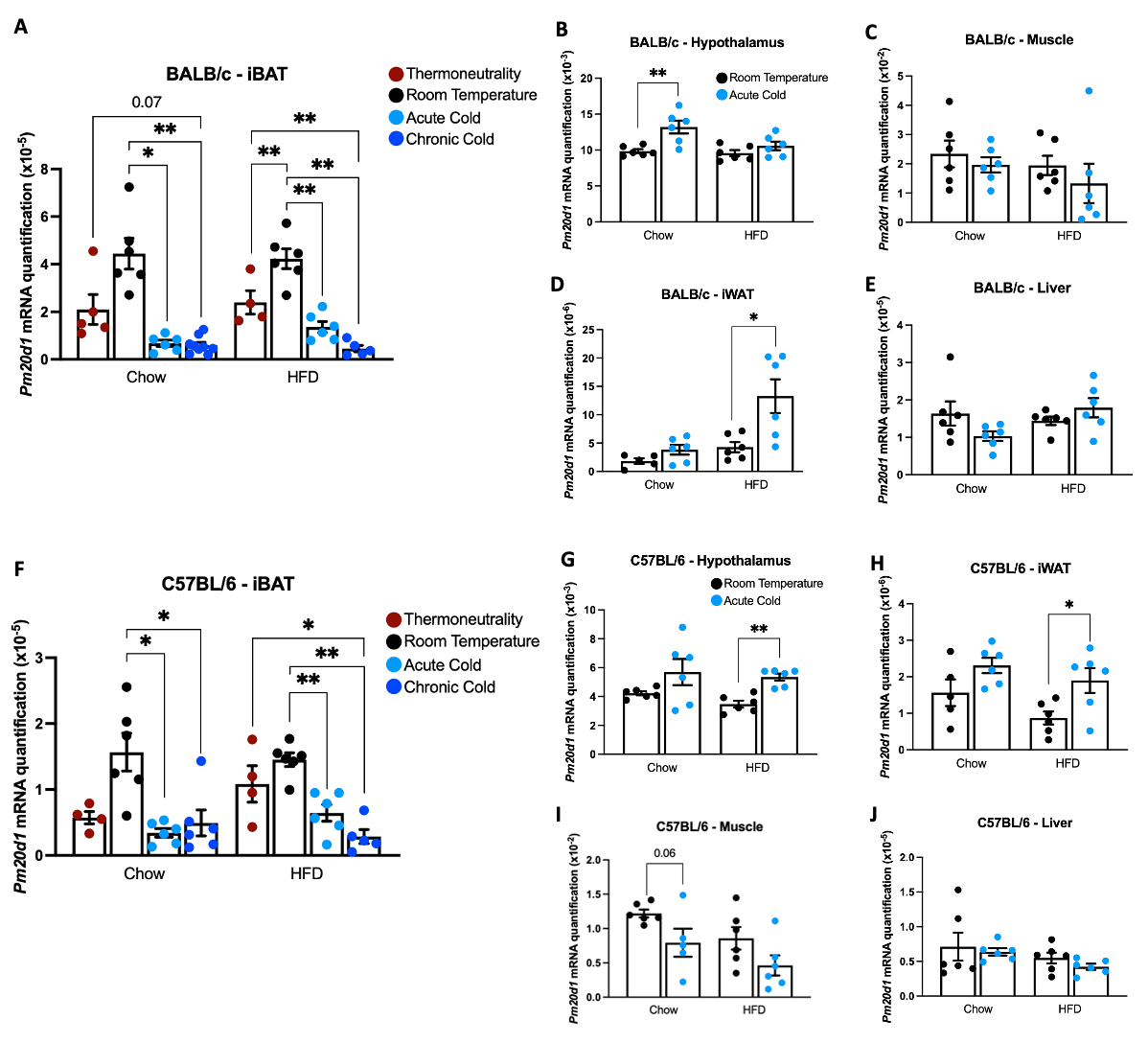
**

**Supplementary Figure 1. *Pm20d1* expression at different temperatures in BALB/c and C57BL/6, across various tissues.** (A and F) Expression in the interscapular brown adipose tissue in BALB/c and C57BL/6, respectively (n=4-9); (B and G) Expression in the hypothalamus in BALB/c and C57BL/6, respectively (n=6); (C and H) Expression in the inguinal white adipose tissue in BALB/c and C57BL/6, respectively (n=5-6); (D and I) Expression in the gastrocnemius muscle in BALB/c and C57BL/6, respectively (n=5-6); (E and J) Expression in the liver in BALB/c and C57BL/6, respectively (n=6). In all: male mice at 11 weeks of age after six hours fasting; values are expressed as mean ± SEM, *p<0.05, **p<0.01, independent samples Student’s T-test or Mann-Whitney test (B-E and G-J), and One-Way ANOVA or Kruskal-Wallis test (A and F). iBAT: interscapular brown adipose tissue; iWAT: inguinal white adipose tissue; HFD: high-fat diet.

**
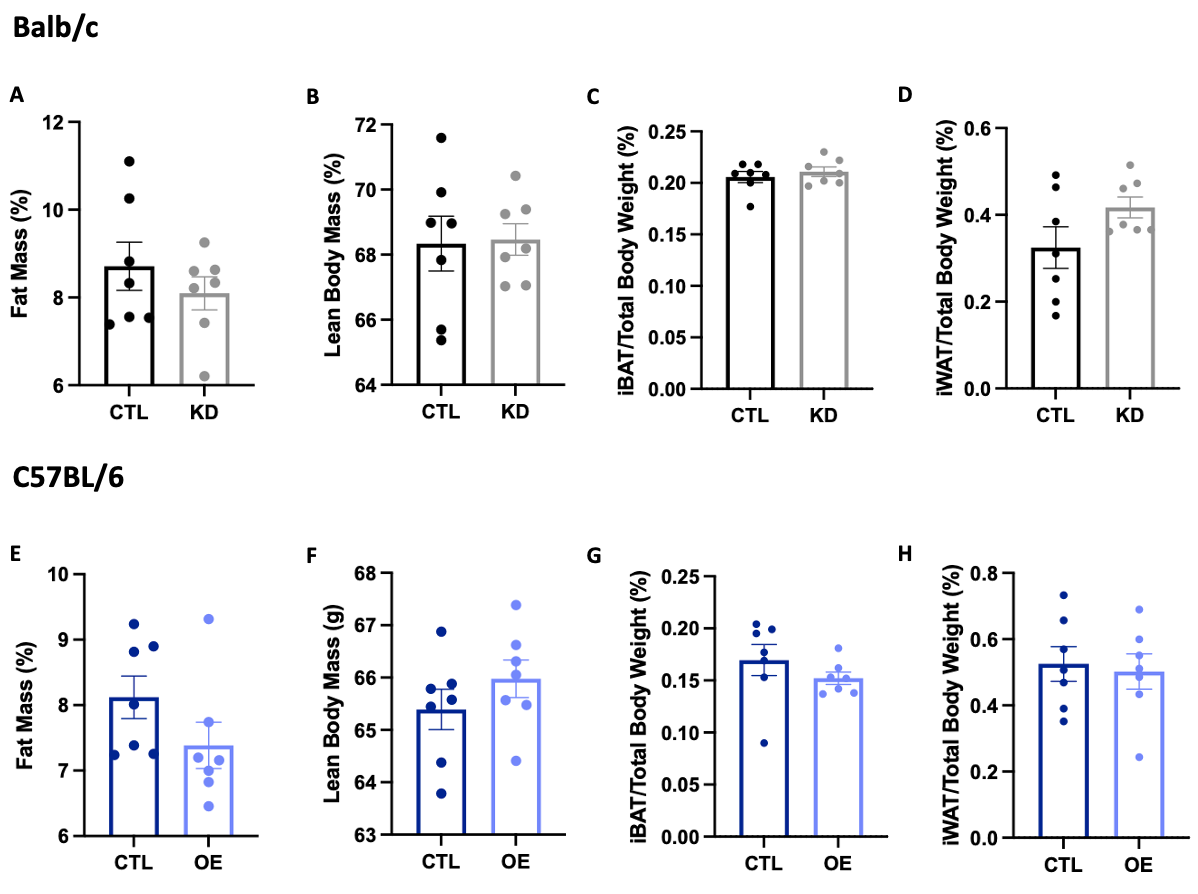
**

**Supplementary Figure 2. Effect of Adeno-associated viral (AAV) injection into iBAT on body composition and adipose tissues.** (A-D) AAV injection to knockdown *Pm20d1* in BALB/c; (A and B) Fat mass percentage and lean body mass percentage, respectively (n=7); (C and D) iBAT weight percentage and iWAT weight percentage, respectively (n=7); (E-H) AAV injection to knockdown *Pm20d1* in C57BL/6; (E and F) Fat mass percentage and lean body mass percentage, respectively (n=7); (G and H) iBAT weight percentage and iWAT weight percentage, respectively (n=7). In A, B, E, and F: male mice at 10 weeks of age; In C, D, G, and H: male mice at 12 weeks of age. In all: values are expressed as mean ± SEM, independent samples Student’s T-test or Mann-Whitney test. CTL: control; KD: knockdown; OE: overexpression.
